# Precursors of Openness to Provide Online Counseling: The Role of Future Thinking, Creativity, and Innovative Behavior of Future Online Therapists

**DOI:** 10.3389/fpsyg.2022.848235

**Published:** 2022-04-01

**Authors:** Dorit Alt, Meyran Boniel-Nissim, Lior Naamati-Schneider, Adaya Meirovich

**Affiliations:** ^1^Faculty of Social Sciences and Humanities, Kinneret College, Jordan Valley, Israel; ^2^Faculty of Education and Instruction, Tel Hai College, Upper Galilee, Israel; ^3^Department of Education, The Max Stern Yezreel Valley College, Yezreel Valley, Israel; ^4^Health Systems Management, Hadassah Academic College, Jerusalem, Israel

**Keywords:** creativity, future thinking, innovation, online counseling, higher education

## Abstract

The outbreak of the COVID-19 pandemic has accelerated the need for online counseling to preserve therapeutic processes that have begun face to face and to provide service to others in need during lockdowns. Previous studies underscored the importance of providing updated training as counselors frequently hesitate to use technological advances in therapeutic sessions. This study aims at reducing such barriers by revealing personal characteristics of future professionals that might inhibit or encourage their openness toward providing online counseling. To this end, this study is focused on several precursors of openness to provide online counseling: preference to communicate emotions online, identification of emotional expressiveness advantages in providing online counseling, innovative behavior, creativity, and future problem-solving thinking skills. The question at focus is which constructs would be found contributive to students’ openness to provide online counseling. The sample included 277 undergraduate students (future counselors) who filled out questionnaires. Data were analyzed using Partial Least Squares Structural Equation Modeling. Our findings pointed to the centrality of students’ preference to communicate their emotions online in explaining their openness to conducting online counseling. This study might help pinpointing the adjustments curriculum designers should address to better reflect the intensive changes within the counseling field that necessitate transferring face-to-face skills to online settings.

## Introduction

The emergence of the COVID-19 pandemic led to imposed social distancing regulations, forcing therapists worldwide to implement an online counseling process through chat, email, or video conference (Situmorang, 2020). This has raised the call to prepare future and contemporary therapists (e.g., educational counselors, social workers, psychologists, healthcare providers, informal educators, etc.) to deliver effective online counseling (Rathenau et al., 2021). In the following innovative study, we investigated several precursors of future counselors’ (meaning students) openness to provide online treatment sessions. The first relates to social-emotional expressiveness, including preferences for online social interaction, and perception of cyber counseling. The importance of this aspect is strengthened in previous studies (Heinonen and Nissen-Lie, 2020) which underscored the importance of social-emotional characteristics such as emotional expressiveness in face-to-face therapy. Therefore, it seems worthwhile to assess the role of emotional aspects in online treatment (Derks et al., 2008). The second precursor is the perceived innovative behavior. Recently, due to the COVID-19 spread, the importance of innovation in online health counseling has been emphasized by researchers (Situmorang, 2020), who also pointed at the dearth of relevant education modules that deal with innovative counseling techniques (Huda and Heroza, 2016). Accordingly, it is important to evaluate how students’ perceived innovation relates to their tendency to provide online counseling. The third precursor is perceived creativity. Creativity is considered a required skill for adaptation in a constantly changing organizational environment and a crucial aspect in providing effective online counseling (Bennett-Levy, 2019). Lastly, awareness of future problems (Cramond, 2009), namely students’ reported tendency to think of future problems and ways to solve them is addressed in the current study, as this skill was found contributive to creativity and innovative behavior (Vijayaratnam, 2012). Hence, this study set out to identify the characteristics of therapists who would be willing to provide online assistance and sought to examine, what characterizes those who are open to providing online counseling and acknowledge its advantages.

Despite the preponderance of the evidence pointing to the advantages of online counseling, providing training for it is only at nascent stages, and therefore, there is scarce literature on training practitioners for online counseling (Anthony, 2015). Moreover, current studies underscore the importance of providing updated training as counselors frequently hesitate to use technological advances in therapeutic sessions (Nagarajan and Yuvaraj, 2019). This study might help reduce such barriers by revealing personal characteristics of future professionals that might inhibit or encourage their openness toward providing online counseling. In addition, the current investigation may help pinpoint the adjustments curriculum designers should address to better reflect the intensive changes within the counseling field that necessitate transferring face-to-face skills to online settings.

## Literature Review

### The Effectiveness of Online Counseling

Health counseling services evolve at a speed that requires appropriate training to keep up with them, thus posing a genuine concern for the profession. This process necessitates transferring face-to-face abilities to online environments (Anthony, 2015). eHealth has emerged in the twenty first century and is an all-encompassing term for the combined use of electronic information and communication technology in the health sector. eHealth can increase networking, facilitate global thinking, and improve health care on the local, regional, and national levels. The apparent advantages of using online interventions include the convenience they allow, overcoming logistical difficulties and approaching populations that avoid seeking treatment (Granja et al., 2018).

eHealth includes online counseling. Mallen and Vogel (2005, p. 764) defined online counseling as “any delivery of mental and behavioral health services, including but not limited to therapy, consultation, and psychoeducation, by a licensed practitioner to a client in a non-face-to-face setting through distance communication technologies, such as the telephone, asynchronous email, synchronous chat, and videoconferencing.” The National Board for Certified Counselors defined online counseling as “the practice of professional counseling and information delivery that occurs when a client and counselor are in separate or remote locations and utilize electronic means to communicate over the Internet” (National Board for Certified Counselors [NBCC], 1997, p. 1).

The unique advantages that the online counseling method has over other counseling methods are those such as better accessibility, an opportunity for self-reflect and self-monitor through written text or watching over again the video, and a sense of control. In addition, the *disinhibition effect* (Suler, 2004) in the online sphere also creates more opportunities for clients to discuss difficult and more complex, sensitive, and personal issues than those discussed in face-to-face counseling, hence showing less worry of the matters of social stigma. Lastly, the negation effect that most people with severe issues face can be mitigated when online counseling is offered in addition to or in place of face-to-face counseling, which may be too intimidating for some (Zainudin et al., 2020).

Despite the existence of advanced and common technologies that enable online counseling as well as studies showing that the effectiveness of online counseling does not diminish compared to face-to-face counseling (Stefan and David, 2013; Glasheen et al., 2016), it seems that counselors, as well as patients, prefer face-to-face counseling. For instance, a recent study on academic students showed that only 35% of participants prefer online counseling over face-to-face counseling (Wong et al., 2018). Another study revealed that counselors choose to provide face-to-face counseling over online counseling. The main reasons to avoid online counseling from counselors’ perspective is lack of training that will allow for dealing with ethical questions, using techniques adapted to the virtual space, and creating tailored skills (Nagarajan and Yuvaraj, 2019).

However, the new reality in light of the eruption of the COVID-19 virus, which forced social distancing, required mental-health professionals to adapt to the new circumstances. With not much time for training and adjustment, they needed to provide their services online. Mental health services were required to be more accessible to the population, enable ongoing treatment, and deal with new health and emotional difficulties that arose in the face of dealing with the new reality. A previous study showed that psychologists who had provided online counseling in the past presented positive attitudes toward online counseling (Cipolletta and Mocellin, 2018). But what about those who didn’t have any experience? What characterizes counselors who have adequately adapted to the changes in the educational and mental healthcare system?

There is a lack of evidence regarding the characteristics of therapists who would be willing to provide their assistance online. Thus, the present study seeks to examine, among future counselors, what characterizes those who are open to providing online counseling and acknowledge its advantages. One of those is emotional expressiveness on online platforms.

### Counselors’ Emotional Expressiveness Online

Heinonen and Nissen-Lie (2020) presented a systematic review of 31 studies identifying influential psychotherapists’ professional and personal characteristics as predictors of patient outcomes. Those comprise, among others, verbal fluency, responsiveness, and emotional expressiveness. The review refers to face-to-face therapy, yet it seems plausible to assume that emotional expressiveness is an essential component of any kind of support or treatment, online or offline. It appears that the conditions that exist online may precede emotional sharing in a way that exceeds that which exists in physical space. For example, an online meeting is usually done in a place comfortable for the patient; current availability allows for quick and immediate sharing; and the symbols and emojis are additional tools for emotional expression (Derks et al., 2008). In this sense, our hypothesis regarding emotional expressiveness is that people who find the online sphere a preferred space for emotional expression will also recognize its benefits as an online therapy platform and, therefore, be more open to providing support online.

H1: Students who prefer communicating their emotions online would tend to see more emotional expressiveness advantages in providing online counseling and would be more open to conducting online counseling.

H2: Students who tend to see more emotional expressiveness advantages in providing online counseling would be more open to conducting online counseling.

### Innovation: Implications for Online Counseling

The Unified Theory of Acceptance and Use of Technology (UTAUT) framework (Venkatesh et al., 2003) offers a comprehensive model of acceptance and successive consumption of technological innovations. The UTAUT was developed to provide a valuable tool to assess the probability of acceptance of new information technology by its users and help clarify the factors that influence the acceptance of new technology, thus enabling educating and training professionals to increase acceptance of technology. Although the UTAUT model was initially developed to be applied in a business environment, it has been adapted to various fields that used technological innovations (Dwivedi et al., 2020). Recently this model was used to evaluate therapists’ attitudes toward online therapy via videoconference (Connolly et al., 2020). According to the original UTAUT model, the use of technology depends on how much the individual believes that using the technology will help them to perform better; the degree of ease associated with the use of the given technology; the extent to which the individual believes that significant others think that they should use the technology; and the perceived level of available professional and technical support in using the technology.

Other studies ascribe the tendency to embrace new services to personal characteristics, specifically to innovative work behavior (IWB) of individual employees (De Jong and Den Hartog, 2010). Drawing on West and Farr (1989) work, Janssen (2000) defined IWB as “the intentional creation, introduction and application of new ideas within a work role, group or organization, in order to benefit role performance, the group, or the organization.” IWB relates to the generation, promotion and implementation of ideas and includes opportunities for adaptation (Zhou and Verburg, 2020). It pertains to an individual’s initiation of new and valuable ideas, products or procedures (Farr and Ford, 1990; De Jong and Den Hartog, 2010). The ability to innovate is deemed crucial for contemporary workplaces, organizations, networks, and individuals. The current research is focused on the level of students in academia and their perceived ability to generate and implement ideas (Bos-Nehles et al., 2017; Bos-Nehles and Veenendaal, 2019).

De Jong and Den Hartog (2010) asserted that an innovation process often starts with a discovery or is triggered by a problem requiring an urgent response. This process leads to idea exploration and finding ways to improve products, services, or processes. Subsequently, ideas are generated, offering solutions to the problems. This process includes finding support by showing enthusiasm and confidence about the innovation, and finally, implementing the concept, testing and modifying it.

Innovative work behavior is conceptualized in the current study as the types of behavior students will engage in to improve their learning or vocational processes. Contemporary students are required to be able to cultivate IWB before entering the work market. Yet IWB studies mainly focused on individual employees, with less attention paid to college students (Aboobaker and Zakkariya, 2019). It has been argued (Etikariena, 2020) that students’ IWB should be fostered earlier to better prepare these prospective workers to deal with the typical demands in the world of work today and the demands displayed during their learning in college. Hence, students should be empowered to be more innovative, a vital skill required also for healthcare practitioners (Radaelli et al., 2014; Oppi et al., 2019; Carlucci et al., 2020). However, despite increased interest in IWB, research remains ancillary in higher education (Etikariena, 2020).

The field of innovation studies in online/cyber health counseling has burgeoned recently due to the COVID-19 outbreak (Situmorang, 2020). Yet, although extensively used during the pandemic, the use of remote health services is not new (Suler, 2001) and has been augmented in the recent decade. Therefore, it seems evident that this trajectory would entail an appropriate preparation of students to effectively use this medium (Mishna et al., 2013). However, despite the growing attention paid to a wide range of innovative techniques in online services (such as video conference) over the last two decades (McCrickard and Butler, 2005), higher education key stakeholders fail to grasp the scope of the phenomenon and to teach students about it. Consequently, contemporary curricula lack relevant education modules that deal with innovative counseling techniques (Huda and Heroza, 2016). In this context, Situmorang (2020) drew attention to the importance of students being able to demonstrate essential innovation aptitudes. In relation to online counseling during the COVID-19 pandemic, the author argued that online counseling services cannot be practiced by practitioners “who do not have the essential innovation aptitudes” (p. 166). Accordingly, in our study, we expect IWB to serve as an essential predictor of online counseling.

H3: Students who report exhibiting innovative behavior would be more open to conducting online counseling.

### Creativity: Implications for Online Counseling

Creativity is defined as “the tendency to generate or recognize ideas, alternatives, or possibilities that may be useful in solving problems, communicating with others, and entertaining ourselves and others” (Franken, 1994, p. 396). In addition, creativity is seen as the ability to produce ideas and products that are innovative, i.e., original or unexpected, and useful, i.e., appropriate, or meet task constraints (Runco and Jaeger, 2012). Creativity is considered a key to success to keep pace with recent changes. It is a required skill for renewal and adaptation in a constantly changing organizational environment and can no longer be considered a luxury (Alt and Raichel, 2018).

IWB researchers often make a distinction between creativity (exploring and generating ideas) and innovation (championing and implementation of ideas) (De Jong and Den Hartog, 2010). IWB differs from employee creativity as it also includes implementing ideas, or an output, intended to provide a benefit. Hence creative individuals will more frequently express new ideas or multidimensional solutions to ill-structured problems, whereas innovative individuals would also act to bring a change about (Etikariena, 2020).

Creative activity has been used to palliate psychological angst and has been recognized as fundamental to the counseling practice (Duffey, 2015; Gladding, 2016). Gladding (2008) asserted that creativity is a crucial aspect in providing effective counseling, “It is through creativity that major theories of counseling and skills in counseling have been developed” (p. 97). Furthermore, creativity bears a sustainable impact on counseling progress in the future. Therefore, it is essential to nurture counselors’ creativity by encouraging them to observe human nature from multiple perspectives.

The associations between creativity and counseling are primarily discussed in previous studies. For example, Patrick (2020) probed the relationship between mistakes, improvisation, and counseling involving a failed clinical intervention. Examination of errors allows assessing diverse elements of counseling while emphasizing the creative nature of the counseling process. The author calls to encourage educators to integrate discussions of mistakes and creative development into training.

Online counseling requires creative thinking to foster an intimate and secure environment that allows clients to freely articulate their thoughts and feelings (Bennett-Levy, 2019). Yet, despite the growing use of cyber counseling, past research mainly discussed the centrality of creativity in online counseling programs rather than exploring actual practices of online counseling. For example, according to Villarreal-Davis et al. (2021), online counseling programs are frequently recognized as less effective due to the lack of face-to-face interaction; however, infusing creative techniques into online programs might facilitate personal growth, enhance professional development, and increase self-awareness for counselors-in-training. The authors proposed using techniques such as expressive arts, online creative writing and pictured miniatures with mindfulness to enhance students’ creativity during online counseling courses. Such innovative approaches may help future counselors become more confident in their counseling skills. Hence, implementing creative strategies in counseling programs may allow students to facilitate effective online counseling. Furthermore, understanding the unique inter-and intrapersonal dynamics of such online sessions during the training period can benefit future practitioners, as well as therapists and counselors that are currently entering the world of online therapy. Therefore, future counselors can incorporate the techniques used during their online counseling training (Pillay et al., 2020).

The present study will examine creativity by assessing the learner’s perception of creativity as an essential element in his/her self-definition (Farmer and Tierney, 2017; Kaufman and Sternberg, 2019). Thus, we will examine the individual’s belief in his/her ability to produce creative ideas and the centrality of this belief in the overall self-description (Plucker and Makel, 2010). Learners who believe in their creativity tend to invest more in creative tasks and effectively solve problems that require creative thinking (Pretz and Nelson, 2017). Furthermore, this belief has been found to be related to creative behavior (Lemons, 2010; Gu et al., 2017; Czerwonka and Karwowski, 2018) and creative achievements (Karwowski and Barbot, 2016). Our study suggests that creative students might be more aware or think of diverse ideas and techniques that can be used in online counseling to provide safe and effective online counseling.

H4: Students who perceive themselves as creative would tend to see more emotional expressiveness advantages in providing online counseling.

Innovation and creativity have been shown to be highly connected to the ability to solve problems, and more specifically, in recent years-so the ability to solve future problems (Treffinger et al., 2021).

### Future Thinking, Creativity, and Innovation

The importance of fostering students’ ability to solve future problems had been underscored in educational programs worldwide during the last two decades (Treffinger et al., 2012; Main et al., 2019). These programs used complex, open-ended problems based on daily life, relevant to the future, and rooted in social contexts. The core issue may address current trends that may develop and affect the human race in the future, and the solution should suggest changing or adapting society to future situations. The problem must be complex and address social, political, business, or technological issues and must take into account future trends that are ingrained in the current era. This inquiry activity invites students to present creative solutions. Students are expected to develop skills that they can apply throughout their lives (Main et al., 2019).

The process of future problem solving (FPS) includes six consecutive steps (Cramond, 2009): (1) Identifying challenges and scenarios that may arise in the future. At this point, many possible issues may arise from the challenge identified. Therefore, students should locate several problems related to the situation and try to obtain information that might help them understand them better, for example, understand the causes of their problems and possible consequences; (2) Selecting a fundamental problem to be addressed. Based on the previous step, learners think of a core problem whose solution may contribute significantly to solving the broader challenge; (3) Generating ideas for solving the underlying problem without judgment. Students think of diverse, unusual, unreasonable and even imagined “crazy” ideas for the chosen problem; (4) Creating and selecting criteria for evaluating the proposed solutions. At this point, students indicate appropriate criteria for evaluating their solutions to determine the best solution. The criteria can be indicators of the quality of the solution, such as safety, efficiency, economics, or morality; (5) Evaluating the solutions. Students evaluate the solutions based on the proposed criteria by ranking each one of them according to each criterion; (6) Development of an action plan, demonstrating how the solution raised to solve the underlying problem will be implemented in practice. This step requires students to consider how they might implement the chosen solution.

Future thinking studies have demonstrated this ability effectiveness in developing creativity and innovation skills (Treffinger et al., 2021). For example, Treffinger et al. (2012) investigated the effectiveness of the program for solving future problems among high school students and showed how the program helped participants develop more vital images of their future and increase their creative abilities. Similarly, Azevedo et al. (2019) have explored an international program for solving future problems to promote adolescents’ creative and critical thinking and future thinking. The results showed the effectiveness of the program in enhancing students’ creative skills compared to a control group.

It should be noted that scant research has been found on the subject that has examined the application of the method and its effectiveness in higher education. For example, Vijayaratnam (2012) showed the advantages of using a futuristic real-world case adapted from the Future Problem Solving website to students’ higher-order thinking, problem-solving, and team skills. Given the dearth of research and lack of in-depth analysis done on the subject in higher education, in the current study, we evaluate students’ tendency to reflect on future problems by using the six components of FPS (Cramond, 2009), and postulate that students’ reported tendency to think of future problems and ways to solve them would positively contribute to their perceived creativity and innovative behavior.

H5: Students who tend to be aware of future problems and ways to solve them would perceive themselves as creative and report exhibiting innovative behavior.

### The Current Study

**FIGURE 1:**
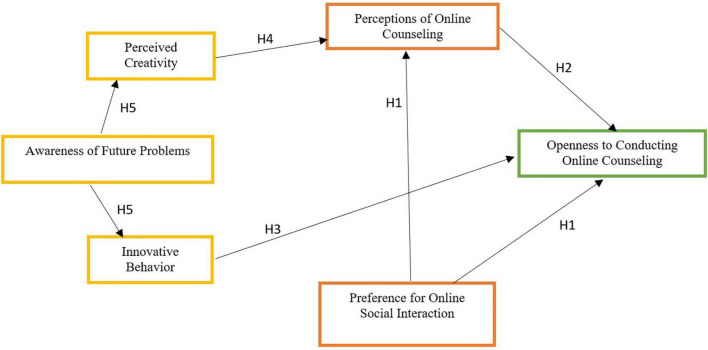
Model 1. The theoretical structure of the proposed framework.

The literature review underscores the advantages of using online counseling interventions and mainly suggests that despite the technologies at hand that enable online counseling (Dunn, 2012; Glasheen et al., 2016), counselors prefer to provide face-to-face counseling over online counseling. Yet, face-to-face counseling might become unavailable during times of enforced social distancing. Therefore, identifying precursors of openness to provide online mental health and medical counseling among future online therapists is of special importance. This study is focused on several precursors of openness to provide online counseling: preference to communicate emotions online, identification of emotional expressiveness advantages in providing online counseling, innovative behavior, creativity, and future problem-solving. The question at focus is which constructs would be found contributive to students’ openness to provide online counseling. Based on the theoretical review, the following hypotheses were formulated (Figure 1 presents the theoretical structure of the proposed framework):

H1: Students who prefer communicating their emotions online (*Preference for Online Social Interaction)* would tend to see more emotional expressiveness advantages in providing online counseling (*Perceptions of Online Counseling*) and would be more open to conduct online counseling.

H2: Students who tend to see more emotional expressiveness advantages in providing online counseling (*Perceptions of Online Counseling)* would be more open to conduct online counseling.

H3: Students who report exhibiting innovative behavior would be more open to conduct online counseling.

H4: Students who perceive themselves as creative (*Perceived Creativity*) would tend to see more emotional expressiveness advantages in providing online counseling (*Perceptions of Online Counseling)*.

H5: Students who tend to be aware of future problems and ways to solve them (*Awareness of Future Problems)* would perceive themselves as creative and report exhibiting innovative behavior.

## Materials and Methods

### Participants

Data were gathered from 277 Israeli third-year undergraduate students, of whom 113 Management of Health Service Organizations students (this program covers patient-doctor relations, quality of service in the healthcare system, and ethics and patient rights). Most of the students in this track are professionals who come from clinical therapeutic fields, are part of the health system and have a background and experience in clinical organizations, such as health counseling. They seek to advance and expand their academic knowledge beyond the therapeutic field to the administrative field. Such an integrated degree enables advancement in the field while having a broad multidisciplinary vision, taking into account systemic considerations. Therefore, the students enrolled in this track are required to develop relevant and specific abilities needed in fields, such as counseling, as part of adapting health systems to the needs of the changing professional requirements.

The sample also included 164 Behavioral Sciences students, aiming to work in therapeutic services with no previous experience in providing social support online. Data included student’s ethnicity, gender, age, and socioeconomic status. Students’ socioeconomic status (SES) was assessed by the father’s educational attainment and the mother’s educational attainment, and both defined on a six-level scale: 0 = *lack of education*, 1 = *elementary school*, 2 = *high school*, 3 = *BA degree*, 4 = *MA degree*, 5 = *doctoral degree*. The mean age of the participants was 24.90 years (SD = 6.20), and 81% were females. The distribution regarding ethnicity was: 59% Jewish students; 39.5% Arab (Muslim, Druze, Christian, and Bedouin) minority students, and 1.5% depicted their religion as “other”. Non-significant differences were indicated between the groups of students on the above variables, except for ethnicity. The Behavioral Sciences group included more minority students (74%) than the Health Management group (35%).

### Measurements

#### Preference for Online Social Interaction

This measurement (Peter and Valkenburg, 2006) includes three items on preference to communicate through the internet on personal issues rather than face to face. Each of the statements was measured on a 5-point Likert scale from 1 = *strongly disagree*, to 5 = *strongly agree*, for example, “on the internet I can talk freely on my emotions than face to face.” The internal consistency of the scale yielded a satisfactory result: α = 0.95.

#### Openness to Conducting Online Counseling

The participants were asked to respond to a single item (Teh et al., 2014), “How would you rate your openness to conducting online counseling?” They were asked to select their answers from the following options coded on a 4-point scale: (1) “I think online counseling is not for me,” (2) “open, but with major reservations,” (3) “open, but with minor reservations,” and (4) “completely open.”

#### Perceptions of Online Counseling Factors

This eight-item scale (Centore and Milacci, 2008; Teh et al., 2014) was designed to measure the participants’ belief that the following components of therapy can be provided by online counseling. It measures the perceived advantages of online counseling. The eight items are: providing a sense of safety and confidentiality, providing empathy, providing connection, accessibility, providing emotional support, availability of counselor, reducing social stigma, and anonymity. Each of the statements was measured on a 5-point Likert scale from 1 = *strongly disagree*, to 5 = *strongly agree.* The internal consistency result was α = 0.90.

#### Perceived Creativity

The *Short Scale of Creative Self (SSCS)* was used to assess this variable. This 11-item scale (Karwowski, 2011, 2014) measures the student’s belief s/he is creative and the belief that creativity is an essential element of her/his overall self-description. Each of the statements was measured on a 6-point Likert scale from 1 = *definitely not*, to 6 = *definitely yes*, for example, “I am sure I can deal with problems requiring creative thinking’ or ‘Being a creative person is important to me.” The internal consistency of the scale was satisfactory: α = 0.93.

#### Awareness of Future Problems

Based on the theoretical framework surveyed above, this scale was constructed for the purpose of the current study. This six-item scale corresponds to the six steps of the FPS program (Torrance and Cramond, 2002). The participants were asked to indicate their level of awareness of social problems that might arise in the future and ways of solving them. Items such as “I think about major social issues that may arise in the future” were scored on a six-point Likert-style format scale (from 1 = *never* to 6 = *always*). The Cronbach’s alpha result was equal to 0.86.

#### Innovative Behavior

This 10-item scale was originally designed by De Jong and Den Hartog (2010) to measure innovative work behavior. For the purpose of this study, the items were elaborated to include innovative behavior in academia in addition to work instantiations, for example, “how often do you search out new working/learning methods, techniques or instruments?” The items were scored on a six-point Likert-style format scale (from 1 = *never* to 6 = *always*). The Cronbach’s alpha result was equal to 0.88.

Table 1 displays the descriptive statistics of the research constructs. Following the general guidelines for skewness and kurtosis [suggesting that if the number is higher than + 1 or lower than –1, then the distribution is skewed, flat or peaked, Hair et al. (2017)], it can be learned that the distributions can be considered normal.

**TABLE 1:** Descriptive statistics of the research constructs.

Construct	Mean	SD	Skewness	Kurtosis
Perceptions of Online Counseling	3.76	0.99	0.09	–0.11
Innovative Behavior	4.23	0.73	0.07	–0.30
Perceived Creativity	4.47	0.85	–0.32	0.02
Preference for Online Social Interaction	3.06	1.40	0.12	–0.74
Awareness of Future Problems	4.10	0.83	–0.38	0.50
Openness to Conducting Online Counseling	2.66	1.00	–0.23	–1.00

### Data Analysis

Data were analyzed using Partial Least Squares Structural Equation Modeling [PLS-SEM; Hair et al. (2017)], advised to be applied in situations where theory is less developed, and if the primary objective of using structural equation modeling is a prediction of target constructs.

### Procedure

The questioners were administered to the participants by research assistants via an online link. Before obtaining participants’ consent, it was explained that the questionnaires were anonymous and acceptable should they choose to submit a partially completed questionnaire or decide not to participate. Finally, participants were assured that no specific identifying information would be processed. The study was preauthorized by the college’s Ethics Committee.

## Findings

**FIGURE 2:**
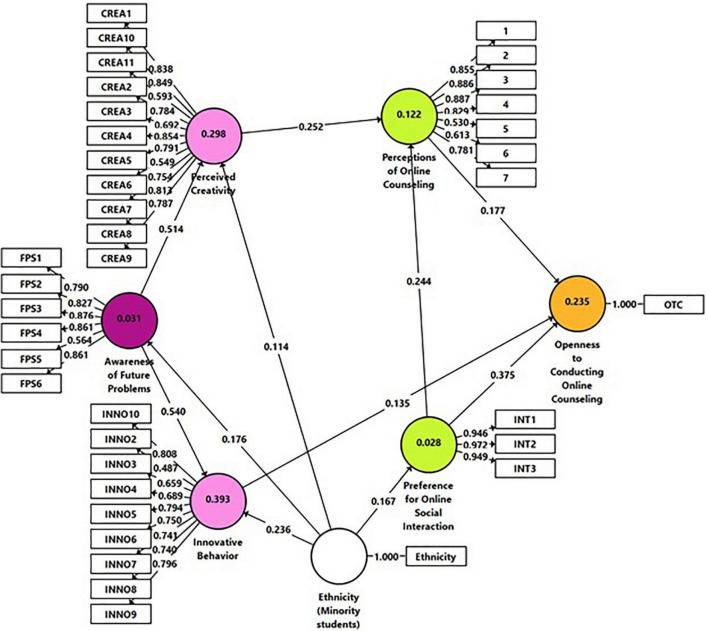
Model 2. Analysis results of the examination of H1-H5 by smartPLS.

To assess H1–H5, Model 2 (Figure 2) was constructed. This path model includes six main constructs, represented in the model as cycles: Awareness of Future Problems, Perceived Creativity, Innovative Behavior, Preference for Online Social Interaction, Perceptions of Online Counseling, and Openness to Conducting Online Counseling. In addition, given the significant between-group differences regarding ethnicity, this variable was entered into the model and paths were specified between this variable and the research factors, yet for visual clarity, the model includes only significant path coefficients detected in the analysis (Gender and Age were also entered into the model to assess how they might impact the research constructs; however, the analysis yielded to non-significant results). As shown in Figure 2, paths were specified based on the proposed hypotheses. Two items were omitted from the model due to low loading results (<0.40).

Table 2 presents the analysis results of the direct and indirect effects. In relation to the direct effects, as shown in the table, Preference for Online Social Interaction exerts a positive effect on Openness to Conducting Online Counseling and Perceptions of Online Counseling. Hence, students who preferred communicating their emotions online also acknowledged more emotional expressiveness advantages in providing online counseling and were inclined toward Openness to Conducting Online Counseling. H1 was confirmed.

**TABLE 2:** Significance analysis of the direct and indirect effects.

Path	Direct effect	*t*-value	*p*-value	Indirect effect	*t*-value	*p*-value
Awareness of Future Problems → Innovative Behavior	0.540	11.876	0.000			
Awareness of Future Problems → Perceived Creativity	0.514	8.246	0.000			
Ethnicity (Minority students) → Awareness of Future Problems	0.176	3.224	0.001			
Ethnicity (Minority students) → Innovative Behavior	0.236	5.028	0.000			
Ethnicity (Minority students) → Perceived Creativity	0.114	2.258	0.024			
Ethnicity (Minority students) → Preference for Online Social Interaction	0.167	2.847	0.005			
Innovative Behavior → Openness to Conducting Online Counseling	0.135	2.459	0.014			
Perceived Creativity → Perceptions of Online Counseling	0.252	4.394	0.000			
Perceptions of Online Counseling → Openness to Conducting Online Counseling	0.177	3.002	0.003			
Preference for Online Social Interaction → Openness to Conducting Online Counseling	0.375	6.593	0.000			
Preference for Online Social Interaction → Perceptions of Online Counseling	0.244	4.294	0.000			
Awareness of Future Problems → Innovative Behavior → Openness to Conducting Online Counseling				0.096	3.053	0.002
Awareness of Future Problems → Perceived Creativity → Perceptions of Online Counseling				0.129	3.988	0.000
Perceived Creativity → Perceptions of Online Counseling → Openness to Conducting Online Counseling				0.044	2.515	0.012
Preference for Online Social Interaction → Perceptions of Online Counseling → Openness to Conducting Online Counseling				0.043	2.284	0.023

It can be learned from Table 2 that Perceptions of Online Counseling increased the levels of Openness to Conducting Online Counseling. Meaning, participants who tended to see more emotional expressiveness advantages in providing online counseling were found more open to conducting online counseling. H2 was approved.

In addition, Innovative Behavior positively contributed to Openness to Conducting Online Counseling. Hence, students who reported exhibiting innovative behavior were more open to conducting online counseling. H3 was corroborated. Perceived Creativity increased the extent of Perceptions of Online Counseling. Thus, students who perceived themselves as creative tended to see more emotional expressiveness advantages in providing online counseling. H4 was approved. Finally, as speculated in H5, both Perceived Creativity and Innovative Behavior were positively informed by students’ Awareness of Future Problems.

As for the indirect effects, the results mainly showed that Awareness of Future Problems indirectly affected Openness to Conducting Online Counseling and Perceptions of Online Counseling by increasing the levels of perceived innovation and creativity, respectively. Moreover, Perceived Creativity contributed to Openness to Conducting Online Counseling by increasing the levels of Perceptions of Online Counseling. Lastly, Preference for Online Social Interaction directly affected Openness to Conducting Online Counseling, but also indirectly by enhanced Perceptions of Online Counseling.

Ethnicity was related to four constructs. Accordingly, minority students reported being more aware of future problems, innovative and creative than majority students, and preferred online social interactions more than their Jewish peers.

### Model Evaluation

Collinearity was examined by the Variance Inflation Factor (VIF) values of all sets of predictor constructs in the structural model. The results showed that the VIF values of all combinations of endogenous and exogenous constructs are below the threshold of five (Hair et al., 2017), ranging from 1.000 to 1.116. Therefore, collinearity among the predictor constructs was not a critical issue in this structural model.

The coefficient of determination (*R*^2^) value was also examined. *R*^2^ for Awareness of Future Problems (0.031) and Preference for Online Social Interaction (0.028) were rather weak. A relatively higher result was indicated for Perceptions of Online Counseling (0.122). Moderate results were shown for Innovative Behavior (0.393), Openness to Conducting Online Counseling (0.235), and Perceived Creativity (0.298).

In addition to measuring the *R*^2^ values, the change in the *R*^2^ value when a specified exogenous construct is omitted from the model was used to evaluate its impact on the endogenous constructs. This measure is referred to as the *f*^2^ effect size when values of 0.02, 0.15, and 0.35, respectively, represent small, medium, and large effects. Small effect size results were indicated between Innovative Behavior and Openness to Conducting Online Counseling (0.023). Small to medium results were shown between Perceptions of Online Counseling and Openness to Conducting Online Counseling (0.037); Preference for Online Social Interaction and Perceptions of Online Counseling (0.068); Perceived Creativity and Perceptions of Online Counseling (0.072). Medium effect size was indicated between Preference for Online Social Interaction and Openness to Conducting Online Counseling (0.173).

Finally, the predictive relevance (*Q*^2^) of the path model was assessed. Values larger than 0 suggest that the model has predictive relevance for a particular endogenous construct (Hair et al., 2017). The highest *Q*^2^ value was indicated for Openness to Conducting Online Counseling (0.214). Lower values were indicated for Innovative Behavior (0.199) and Perceived Creativity (0.168). The lowest results were found for Perceptions of Online Counseling (0.068), Preference for Online Social Interaction (0.021), and Awareness of Future Problems (0.016).

## Discussion

This study sought to assess several precursors of openness to provide online counseling: preference to communicate emotions in online social interactions, identification of emotional expressiveness advantages in providing online counseling, innovative behavior, creativity, and future problem-solving. Our findings pointed to the centrality of students’ preference to communicate their emotions online in explaining their openness to conducting online counseling. This could be explained by researchers such as Stephen et al. (2014), who argue that online communication with patients includes features of text-only interaction. Although sentences are often short in live chat conversations, writing provides time to compose thoughts, reflect on what the participant wanted to convey, and thus elaborate ideas more fully. The authors suggest that the live text-only modality outweighs spoken communication, giving participants time to reflect and compose their thoughts. In the context of the present work, it might be plausible to infer that students who are more able to convey their emotions in social interaction would feel less deterred to use this skill during future online treatment sessions. In addition, this reflective skill can be honed during therapist training programs. They should be encouraged to reflect in writing on the implications of their self-practice for themselves and how to transfer emotional reactions (Bennett-Levy, 2019).

Another finding showed that participants who tended to see more emotional expressiveness advantages in providing online counseling were found more open to conducting online counseling. While emotions are expressed linguistically and para linguistically through non-verbal signs and gestures in face-to-face communication, emotional expressiveness online may include a wide range of visual features. It has, for example, emoji and stickers, by which emotions and feelings can be expressed, thus making the sentiment of the message more salient (Zhao, 2019; Gantiva et al., 2020). It might be inferred that those who can use this visual language to communicate their emotions online effectively might also acknowledge the benefits of these practices in online counseling, thus feeling more capable of providing online sessions that often involve emotional communication.

Another interesting finding showed that students who reported exhibiting innovative behavior were more open to conducting online counseling. Both these constructs reflect behavioral aspects, innovative behavior and online counseling provision. Our findings may imply that the participants’ readiness for change affected their openness toward online treatments. Although not new, they gained increased popularity during recent frequent lockdowns over the more acceptable face-to-face counseling. As stated by Alt and Raichel (2018), situations that require innovation are those in which existing models or solution strategies are unavailable or unproductive, as was the case for face-to-face counseling due to government restrictions imposed during the pandemic.

Our empirical model results also indicated that students who perceive themselves as creative tended to see more emotional expressiveness advantages in providing online counseling. Consequently, they were more open to providing online counseling. Creative individuals generate new and diverse insights or solutions to ill-structured problems, identify the unknown, and seek clarity (Mumford et al., 2009). In addition, creativity often pertains to the ability to produce multiple solutions to a problem by making unexpected combinations or recognizing links among remote associates (Ritter and Ferguson, 2017). This perceived ability for divergent thinking might explain the participants’ belief that multiple components of therapy can be provided by online counseling.

Innovation might be perceived as a personal characteristic within the individual (Etikariena and Widyasari, 2020). Yet, our study adds to the corpus of knowledge by showing how this behavior can be prompted by students’ enhanced awareness of future social problems and ways to solve them. This goes in accordance with Joachim et al.’s (2020) premise that unconstrained and futuristic thinking is a critical mindset in the pursuit of innovation. Furthermore, creativity was similarly influenced by the participants’ awareness of future problems. This finding may point to the benefits of student awareness of social challenges and their perceived ability to offer solutions, evaluate their quality, and devise an action plan to implement their perceived creativity and innovative behavior (Treffinger et al., 2021).

### Limitations and Directions for Future Research

The present work features limitations and directions for future research that warrant mention. Self-reporting measures were used in this study. Some studies find substantial biases in such measurements and strong divergence between subjective and objective assessments; thus, data gained by such measures should be interpreted (Bowman, 2010). However, it is noteworthy that students’ perceptions, attitudes, and beliefs play a central role in their learning and are related to motivation to engage in a specific learning activity (Bandura, 1997). For example, in the context of the current study, students’ perceived willingness to engage in online counseling may drive their future actions.

In addition, based on the low-moderate results of *R*^2^, *f*^2^, and *Q*^2^ our findings must be interpreted with caution. Although a moderate *R*^2^ result was obtained for openness to conducting online counseling (mainly explained by Preference for Online Social Interaction), future research should consider expanding the model tested here with additional variables that could explain this dependent variable. For example, concerning the creativity construct, it would be interesting to explore, for instance, group vs. individual approaches to creativity (Cortini et al., 2019). Creativity in workplace can be viewed as individual or collective, as it can depend on individual traits or rely on sharing attitudes in a group and the common objective or motivation (Cirella, 2016). It would also be interesting to explore how participants’ past experiences with online interactions (e.g., past counseling experiences or daily habits with online communications), may affect the constructs measured in the empirical model. It is also advisable to address other models and tools, such as the UTAUT framework (Venkatesh et al., 2003), to assess the probability of acceptance of new information technology.

Moreover, the sample comprised 81% female students. Although found non-significantly related to the variables of the empirical model assessed in this study, gender differences in emotional expressiveness are extensively documented in previous studies, which analyzed how males and females express their emotions online. For example, Parkins (2012) found that women use emotional expression markers more than men to help display their feelings online. Although, indeed, undergraduate research pools can be overrepresented by women (Dickinson et al., 2012), future studies need to find ways to mitigate gender imbalances by increasing the number of male participants. However, it may come at the cost of non-representativeness.

## Conclusion and Practical Implications

Online communication has been recognized as the best practice for counseling during the social distancing periods imposed due to the COVID-19 outbreak (Situmorang, 2020). Such online communication requires viable expressive abilities in the process, such as utilizing images, portraying sentiments, and using body language. With the growing attention paid to online treatment, future counselors must be introduced to cyber counseling, and their teachers ought to encourage and prepare them to realize the benefits of this medium.

Our study shows that some students already feel comfortable using online platforms to provide counseling services. Those are characterized by thinking of future problems, holding creative and innovative perceptions, and tending toward online social interaction. These abilities and proclivities should be further honed through programs of recurrent practice in cyber counseling. Curriculum designers should consider focusing attention on developing activities to effectively encourage future counselors to use the internet for online treatment. Traditional core training should be updated to include best practices that deal with the concept of cyberculture. In this context, we propose implementing future problem-solving techniques to help students, as well as therapists and counselors that are currently entering the world of online therapy, pinpoint the difficulties they might encounter as providers of online treatment sessions and potential ways to mitigate them. For example, how to portray sentiments or use body language to enhance viable communication. Such future thinking practices were proved useful to increase learners’ creativity and innovation (Treffinger et al., 2021), and as shown in the current study, these outcomes might have students recognize the advantages of online counseling.

## Data Availability Statement

The datasets presented in this study can be found in online repositories. The names of the repository/repositories and accession number(s) can be found in the article/supplementary material.

## Ethics Statement

The studies involving human participants were reviewed and approved by Kinneret college on the Sea of Galilee. The patients/participants provided their written informed consent to participate in this study.

## Author Contributions

DA: conceptualization, data curation, methodology, and writing—original draft preparation, reviewing, and editing. MB-N: conceptualization, data curation, and writing—original draft preparation. LN-S: data curation, methodology, and writing—original draft preparation. AM: data curation. All authors contributed to the article and approved the submitted version.

## Conflict of Interest

The authors declare that the research was conducted in the absence of any commercial or financial relationships that could be construed as a potential conflict of interest.

## Publisher’s Note

All claims expressed in this article are solely those of the authors and do not necessarily represent those of their affiliated organizations, or those of the publisher, the editors and the reviewers. Any product that may be evaluated in this article, or claim that may be made by its manufacturer, is not guaranteed or endorsed by the publisher.
